# Terahertz Optical Properties and Carrier Behaviors of Graphene Oxide Quantum Dot and Reduced Graphene Oxide Quantum Dot via Terahertz Time-Domain Spectroscopy

**DOI:** 10.3390/nano13131948

**Published:** 2023-06-27

**Authors:** Seunghyun Song, Hyeongmun Kim, Chul Kang, Joonho Bae

**Affiliations:** 1 Department of Physics, Gachon University, 1342 Seongnamdaero, Sujeong-gu, Seongnam 13120, Republic of Korea; songsh139@gmail.com; 2 Department of Physics, Chonnam National University, 77 Yongbong-ro, Buk-gu, Gwangju 61186, Republic of Korea; u00extreme@naver.com; 3 Advanced Photonics Research Institute, Gwangju Institue of Science and Technology, 123 Cheomdangwagi-ro, Buk-gu, Gwangju 61005, Republic of Korea

**Keywords:** graphene oxide quantum dot, reduced graphene oxide quantum dot, terahertz time-domain spectroscopy, Drude–Lorentz oscillator model, optical property, carrier behavior

## Abstract

Graphene quantum dots (GQDs) with a band gap have been widely applied in many fields owing to their unique optical properties. To better utilize the optical advantages of GQDs, it is important to understand their optical characteristics. Our study demonstrates the optical properties and carrier behaviors of synthesized graphene oxide quantum dot (GOQD) and reduced graphene oxide quantum dot (rGOQD) pellets via Terahertz time-domain spectroscopy (THz-TDS). The complex permittivity and optical conductivity are obtained in the terahertz region, indicating that the optical conductivity of the GOQD is higher than that of the rGOQD. Although rGOQD has a higher carrier density, approximately 1.5-times than that of GOQD, the lower charge carrier mobility of the rGOQD, which is obtained using Drude–Lorentz oscillator model fitting contributes to a decrease in optical conductivity. This lower mobility can be attributed to the more significant number of defect states within the rGOQD compared to GOQD. To the best of our knowledge, our study initially demonstrates the optical property and carrier behaviors of GOQD and rGOQD in the THz region. Moreover, this study provides important information on factors influencing carrier behavior to various fields in which carrier behavior plays an important role.

## 1. Introduction

Carbon materials have been extensively studied and applied in a wide range of fields in recent decades due to their exceptional properties. These materials exhibit high chemical and mechanical stability, along with excellent electrical conductivity and low toxicity. Graphene, a popular carbon nanomaterial, has semi-metallic properties because of its zero band-gap energy [1,2]. It is widely known that the conduction band and valence band contact at the Dirac point in the energy band structure of graphene. Essentially, the photoluminescence phenomenon by the charge transition in energy levels cannot be observed in graphene. However, its band gap is opened when the size of the graphene decreases to several nanometers, owing to the quantum confinement effect. The effect is that the electrons are confined, leading to quantized energy when the particle size is smaller than the exciton Bohr radius. Graphene quantum dots (GQDs), which are graphene pieces smaller than 20 nm, also have a nonzero band-gap property owing to the quantum confinement effect, exhibiting the photoluminescence property [3,4]. Their fluorescence characteristics make GQDs highly applicable in photo-related fields, such as fluorescent sensing and bioimaging. Furthermore, a significant advantage of the GQDs lies in their tunable band gap, which can be controlled by manipulating their size and functionalization. This property provides significant benefits in various fields that require energy-gap engineering, such as solar cells and photocatalysis [5,6,7]. In these applications, one of the important factors is the charge carrier behavior of material, such as carrier lifetime and mobility, to enhance the efficiency [8,9]. Therefore, the carrier behavior of GQDs needs to be explored as a promising material for various application fields that rely on carriers.

Terahertz (THz) spectroscopy is a useful tool in numerous fields using a terahertz pulse of 100 GHz to 10 THz. It is performed using a noncontact and nondestructive method with low photon energy [10,11,12]. Moreover, it is a highly suitable method for investigating the molecular and carrier dynamics of materials. This is due to the fact that molecular dynamics, such as the vibration and rotation of molecules, has energy levels similar to those of THz waves [13,14,15]. Additionally, it has been used in medical imaging as it poses no harm to the human body, ensuring safety during the imaging process [16]. Among the various THz spectroscopy methods, THz time-domain spectroscopy (THz-TDS) enables exploring the optical properties of materials by measuring the THz pulse containing the information for the material [17]. THz-TDS can obtain the phase and amplitude of a pulse passing through a material directly compared with Fourier-transform infrared spectroscopy (FT-IR) which uses Kramers–Kronig analysis. As a result, optical properties obtained through THz-TDS measurements exhibit smaller errors compared to those obtained using FT-IR analysis [18]. In THz-TDS, time-domain result signals containing measured information are obtained initially, and frequency-domain results can be acquired via fast Fourier transform (FFT). Optical characteristics, including absorption coefficient and complex refractive index in the THz region, can be investigated from the results of THz-TDS measurement. The experimental complex permittivity and AC conductivity of a material can be determined by calculating them using optical constants, and those are analyzed using theoretical modeling, such as Drude model, Lorentz model, and Drude–Smith model [19,20].

In our work, graphene oxide quantum dots (GOQDs) were synthesized using the oxidative cutting method by oxidizing graphite with sulfuric acid and nitric acid. Subsequently, reduced graphene oxide quantum dots (rGOQDs) were prepared through the thermal reduction of GOQDs using a furnace. The size of the prepared GQODs and rGOQDs was approximately 5 nm. The reduction in the quantity of oxygen functional groups within the rGOQDs was verified through the utilization of FT-IR spectrometry and X-ray photoelectron spectroscopy. THz-TDS measurement was conducted to investigate the optical properties of the prepared GOQD and rGOQD freestanding pellets in a frequency range of 0.2 to 2.0 THz. THz absorption was confirmed after the THz waves passed through both samples. Optical parameters, including the absorption coefficient and index of refraction, were obtained from THz-TDS results. The complex permittivity and optical conductivity were calculated using the acquired optical parameters and fitted using the Drude–Lorntz model. Moreover, Drude and Lorentz fitting parameters related to the carrier behaviors for the GOQD and rGOQD were obtained. Finally, Raman spectroscopy was conducted on both samples to verify the THz-TDS results. To the best of our knowledge, our study initially reports the optical property and carrier behaviors of GOQDs and rGOQDs in the THz region. Furthermore, this finding helps pave the way for further research and development in utilizing GQDs for various technological applications by contributing to the advancement of knowledge and understanding in the field of GQDs. 

## 2. Materials and Methods

### 2.1. Preparation of GOQDs and rGOQDs

To synthesize the GOQDs, 0.3 g of graphite powder (US Research Nanomaterials, Inc., Houston, TX, USA) was used as a precursor, which was mixed with 20 mL of HNO_3_ (Merck, Rahway, NJ, USA) and 60 mL of H_2_SO_4_ (Daejung Chemicals & Metals Co., Ltd., Siheung-si, Republic of Korea) (volume ratio, 1:3) for oxidizing. The mixture solution was subjected to ultrasonication for 2 h to ensure uniform dispersion and facilitate the oxidation process. The prepared acidic solution was then stirred at 130 °C for 24 h and subsequently cooled down to room temperature. To neutralize the solution, sodium carbonate (FUJIFILM Wako Pure Chemical Corporation, Tokya, Japan) was added to the solution after adding 80 mL of deionized water. In order to remove impurities that are formed during the synthesis process from the solution, the fractional crystallization method was employed as the initial purification step. Upon cooling down the solution in an ice bath, the solubility of Na_2_SO_4_ and NaNO_3_ salts decreased, causing them to separate from the prepared solution as solid precipitates. As a second step for purification, dialysis was conducted for 7 d using a 2000 MWCO (molecular weight cut-off) dialysis bag. Finally, the GOQD powder was obtained through freeze-drying method. In order to prepare the rGOQDs, GOQD powder was annealed at 400 °C for 2 h under Ar gas flow (80 cc/min) in a horizontal quartz tube furnace. After cooling down to room temperature, rGOQD powder was obtained. 

### 2.2. Materials Characterization

The structural morphologies of the GOQD and rGOQD were observed using transmission electron microscopy (TEM) using an FEI Titan G2 Chemi STEM Cs Probe (Hillsboro, OR, USA). Ultraviolet–visible (UV–vis) absorption spectra were obtained via Varian Cary 50 UV–vis spectrophotometer (Santa Clara, CA, USA). To confirm the removal of oxygen functional groups in rGOQD after thermal reduction, FT-IR spectroscopy was conducted using a PerkinElmer L1600300 Spectrum Two Lita (Waltham, MA, USA) instrument. X-ray photoelectron spectroscopy (XPS) utilizing the Al-Kα radiation of a Thermo Fisher Nexsa (Waltham, MA, USA) spectrometer was also employed. Raman spectroscopy was performed using a 785 nm laser on Renishaw inVia reflex Raman (Wotton-under-Edge, UK) spectrometer to investigate the degree of defects in the samples.

### 2.3. Terahertz Time-Domain Spectroscopy (THz-TDS) Measurement

**Figure 1:**
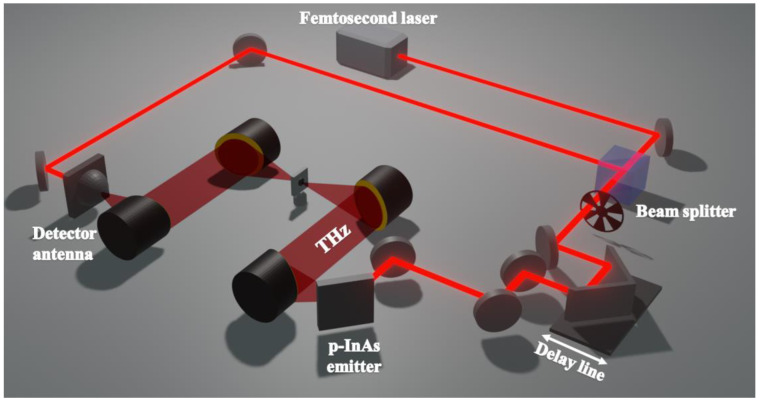
Schematic of the terahertz time-domain spectroscopy experimental setup.

GOQD and rGOQD pellets were fabricated to perform the THz-TDS measurements. To prepare the pellets, the GOQD and rGOQD powders were pressed under a pressure of 20 MPa for 20 min using an 8 mm pellet die. To enhance accuracy and reliability, three pellets were prepared for both GOQD and rGOQD using the same conditions. THz-TDS measurements were carried out three times on all fabricated pellets. The acquired results from each pellet were averaged to provide representative values in order to minimize potential variations or inconsistencies that may arise from individual pellet preparations and THz-TDS measurements. The average thicknesses of GOQD and rGOQD pellets were 225 and 231 μm, respectively. To prevent the absorption of the THz beam by water vapor, the THz-TDS setup was positioned within an acrylic box that was filled with dry air. Figure 1 illustrates the THz-TDS experimental setup. Initially, the Ti-sapphire femtosecond laser pulse was divided into two beams by the beam splitter. One laser beam was guided to the p-InAs wafer, which generates THz waves via the photo-Dember effect. Simultaneously, the other weaker laser beam was guided to a photoconductive antenna on LT-GaAs, which is used to detect THz waves. The optical properties of the sample were obtained by measuring the transmitted THz signal, both with and without the sample. A Ti-sapphire femtosecond laser with an average power of 1.3 W was used at a center wavelength 800 nm with a repetition rate of 79 MHz and pulse width of less than 100 fs.

## 3. Results and Discussions

**Figure 2:**
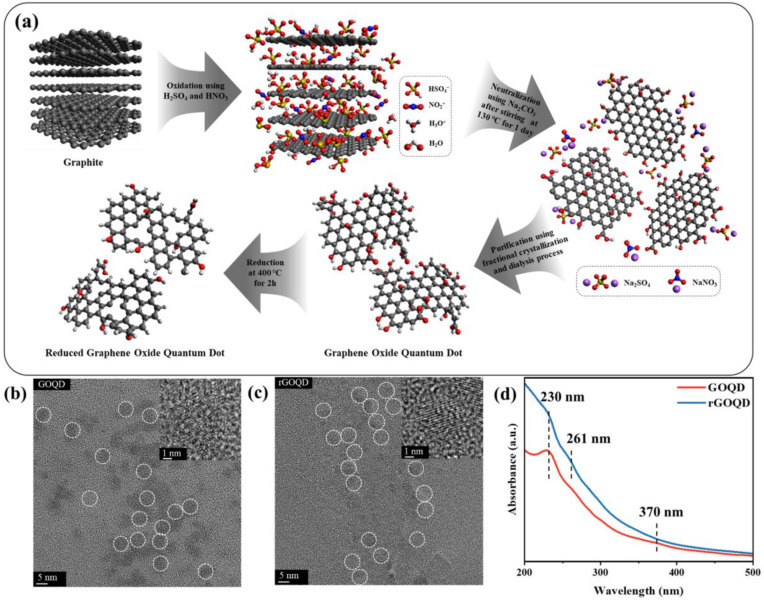
(**a**) Illustration of the synthesis process of GOQDs and rGOQDs by oxidation cutting of graphite. TEM images of GOQDs (**b**) and rGOQDs (**c**). (**d**) UV–vis spectra of GOQDs and rGOQDs.

Figure 2a shows the synthesis process of GOQDs and rGOQDs. To prepare the GOQDs, the oxidative cutting method was used using sulfuric acid (H_2_SO_4_), nitric acid (HNO_3_), and graphite. In a mixture solution of acids and graphite, the oxidation process leads to the expansion of the interlayer spacing in the carbon layers in graphite and the introduction of oxygen functional groups. Subsequently, thermal treatment causes a decrease in the size of the carbon structure, owing to both the breaking of weakened carbon layers and the cleavage of carbon–carbon bonds. rGOQD powder was fabricated using the thermal reduction of GOQD under Ar gas conditions. The detailed explanation of sample preparation is written in the Experimental section. Figure 2b,c show the TEM images of the GOQDs and rGOQDs, respectively. The GOQDs and rGOQDs exhibited a relatively uniform size distribution. The insets of (b) and (c) are the corresponding images of the individual GOQDs and rGOQDs. It exhibits that the diameters of both materials are about 5 nm. The TEM results indicate the successful synthesis of GOQDs and rGOQDs via oxidative cutting. Figure 2d shows the UV–vis absorption curves of the prepared samples dissolved in deionized water. The spectrum of GOQDs exhibited two distinct peaks at 230 nm and 370 nm, corresponding to the π–π* transition of C=C bonds and n–π* transition of C=O, respectively [21]. After the reduction process, the π–π* transition peaks were observed at 230 nm and 261 nm. The redshift of the peaks represents the restoration of the aromatic structure by reduction. Additionally, the peak corresponding to the n–π* transition of C=O bonds was absent in the UV–vis absorbance spectrum of rGOQDs, exhibiting the removal of oxygen-containing groups [22,23].

**Figure 3:**
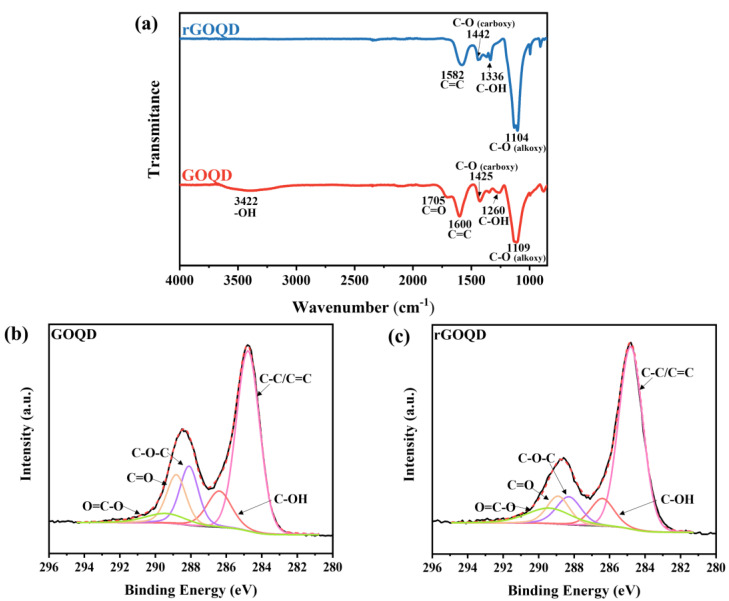
(**a**) FT-IR spectra and XPS C1s spectra deconvoluted peaks of GOQDs (**b**) and rGOQDs (**c**).

FT-IR was performed to examine the change in carbon–oxygen bonding after the reduction of the GOQD. In the FTIR spectrum of the GOQDs, characteristic peaks corresponding to various oxygen functional groups were observed at 3422 cm^−1^ (-OH), 1705 cm^−1^ (C=O), 1425 cm^−1^ (C-O), 1260 cm^−1^ (C-OH), and 1109 cm^−1^ (C-O), as shown in Figure 3a [24]. However, the FT-IR result of rGOQDs indicates a decrease in the oxygen functional group-related peaks, thus verifying the successful reduction of GOQDs. Peaks at 1442 cm^−1^ (C-O), 1336 cm^−1^ (C-OH), and 1104 cm^−1^ (C-O) were observed, whereas those corresponding to the -OH stretching vibration of the COOH functional group and C=O stretching of the carbonyl group disappeared [25,26].

To further investigate the chemical bonding of materials, X-ray photoelectron spectroscopy (XPS) was conducted. Figure 3b,c show the XPS C1s spectra with deconvoluted peaks of the GOQDs and rGOQDs, respectively. The C1s curves were deconvoluted into five different peaks after the binding energy of the XPS spectra was calibrated by setting the peak of C1s at 284.8 eV. The peaks identified by deconvolution correspond to carbon–carbon (C-C/C=C) and carbon–oxygen bonds (C-OH, C-O-C, C=O, and O=C-O) [27,28]. After the reduction process, oxygen-bonding-related peaks decreased compared with those of the GOQDs, as shown in Figure 3c, indicating that it is in agreement with the FT-IR results [29,30]. Moreover, the intensity of peak related to oxygen functional groups at 288.5 eV decreased in the C1s spectrum of rGOQDs. Thus, the FT-IR and XPS results confirmed that the rGOQDs were synthesized successfully using the thermal reduction process.

**Figure 4:**
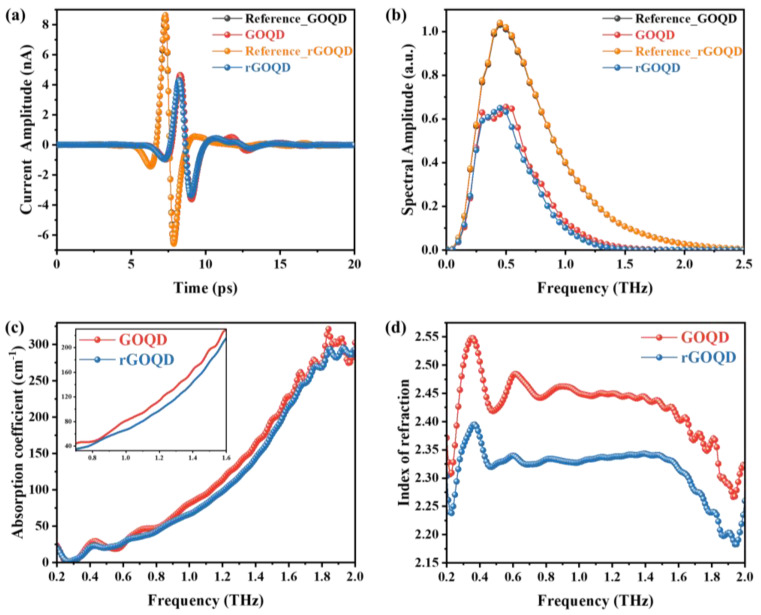
(**a**) THz-TDS measurement result. Time-domain THz wave forms of reference (air), GOQDs, and rGOQDs. (**b**) Frequency-domain signal spectra by FFT. Optical properties of GOQDs and rGOQDs. (**c**) Absorption coefficient *α*(*ω*) and (**d**) index of refractive *n*(*ω*) in the frequency range from 0.2 to 2.0 THz.

To study the optical properties of the GOQDs and rGOQDs in the THz region, THz-TDS was conducted by comparing the transmitted THz signals with and without the sample. The transmitted THz pulses obtained from the GOQDs and rGOQDs are shown in Figure 4a. The THz signal through empty air without the sample was used as a reference signal, and signals passing through the freestanding pellets of GOQDs and rGOQDs were used as sample signals. As shown in Figure 4a, the sample signals of the GOQDs and rGOQDs were delayed and decreased evidently compared with the reference signal in the time domain. The transmitted THz signal of the GOQDs was slightly more delayed and exhibited a larger amplitude due to small absorption compared with that of the rGOQDs. The transmitted THz amplitude of the GOQDs was about 45.1% of the reference signal, whereas that of the rGOQDs was about 49.6%. Figure 4b shows the Fourier-transformed THz amplitude signals of the reference and the samples in the frequency domain. As shown in the curves, the spectral amplitude of both references extended from 0.1 to 2.5, whereas that of GOQD and rGOQD data extended from 0.1 to 1.8 and from 0.1 to 1.6, respectively. This indicates that both samples absorbed a lot of THz pulses in the high-frequency region.

Figure 4c,d show curves of experimental optical constants of the GOQDs and GOQDs, which were calculated from the THz signals measured using THz-TDS. The absorption coefficient *α*(*ω*) and refractive index *n*(*ω*) were obtained using the following equation [31,32].

(1) $$S_{THz\_output}\left(\omega \right)=S_{THz\_input}\left(\omega \right)\exp\left[-\frac{d}{2}\alpha \left(\omega \right)\right]\exp\left[i\frac{2\pi d}{\lambda }n\left(\omega \right)\right]$$

where $S_{THz\_output}\left(\omega \right)$ is the output signal after passing the sample; $S_{THz\_input}\left(\omega \right)$ is the input reference signal; d is the thickness of the sample; and λ is the free space wavelength.

As shown in Figure 4c, the absorption coefficients of both samples increased as the frequency increased from 0.2 to 2.0 THz, exhibiting that the rGOQDs had lower absorption than GOQDs while they had similar values below 0.62 THz. To highlight the disparity in the absorption coefficients between the two samples, the inset graph focuses on the range of 0.7 to 1.6 THz. This difference in absorption coefficients is attributed to the variations in free carriers and Lorentz terms following the reduction of the sample. These changes are clearly illustrated in the inset graph, providing a distinct visual representation of the discrepancy between the two samples. It indicates that the rGOQD sample has more carriers or higher carrier mobility than that of GOQDs. The indices of refraction, the real parts of the complex refractive index, are displayed in Figure 4d. Over the entire measured frequency range, GOQDs had a higher value than rGOQDs with a similar tendency. In the range of 0.2–1.5 THz, the refraction index of both materials exhibited frequency-independent behavior while maintaining similar value levels. However, the refractive indices gradually decreased above 1.5 THz. The oscillations observed at low frequencies in Figure 4c,d can be attributed to multiple reflections caused by the etalon effect. These reflections give rise to interference patterns, leading to the observed oscillatory behavior.

**Figure 5:**
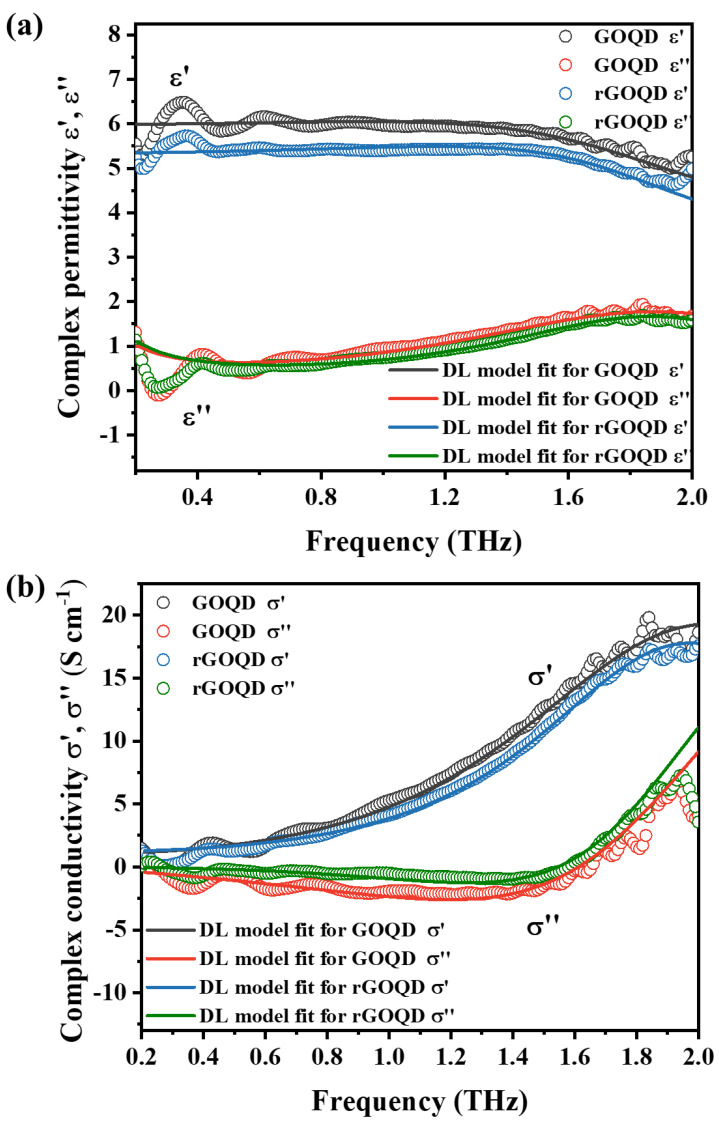
(**a**) Complex permittivity and (**b**) complex optical conductivity of GOQDs and rGOQDs investigated using THz-TDS measurement and fitted results by Drude -Lorentz model (solid line).

Figure 5 shows the real and imaginary parts of the complex permittivity and optical conductivity of the GOQD and rGOQD samples measured using THz-TDS measurement in a frequency range of 0.2–2.0 THz. Moreover, the fitted line from the Drude–Lorentz model also displays in graphs with experimental results. The good agreements between the experimental results and the fitting data indicate the high reliability of the Drude–Lorentz fitting parameters. Furthermore, this agreement suggests that the DL model was properly adopted to fit and analyze the optical properties of the GOQDs and rGOQDs. The prepared GOQDs and rGOQDs have conductive properties similar to those of carbon materials and dielectric properties arising from the oxygen functional groups owing to their bounded charges. Thus, the Drude and Lorentz model was used to analyze the behavior of free and bounded carriers, respectively [33]. The measured complex permittivity and conductivity curves of the samples were obtained using the acquired optical constants. The complex permittivity $\tilde{\varepsilon }\left(\omega \right)=\varepsilon^{\prime }+i\varepsilon^{\prime \prime }$ ( $\varepsilon^{\prime }$ and $\varepsilon^{\prime \prime }$ are real and imaginary part of complex permittivity, respectively) was expressed in terms of *n*(*ω*) and κ (*ω*) (the extinction coefficient *κ*(*ω*); the imaginary part of the refractive index was obtained by *α*(*ω*) = 4*πκ*(*ω*)/*λ*), as follows [19,34].

(2) $$\tilde{\varepsilon }\left(\omega \right)={\left\{\tilde{n}\left(\omega \right)\right\}}^{2}$$

(3) $$\varepsilon^{\prime }=n{\left(\omega \right)}^{2}-\kappa {\left(\omega \right)}^{2},\;\varepsilon^{\prime \prime }=2n\left(\omega \right)\;\kappa \left(\omega \right)$$

The complex conductivity $\tilde{\sigma }\left(\omega \right)=\sigma^{\prime }+i\sigma^{\prime \prime }$ ( $\sigma^{\prime }$ and $\sigma^{\prime \prime }$ are real and imaginary part of complex conductivity, respectively) was calculated as follows.

(4) $$\tilde{\sigma }\left(\omega \right)=-i\omega \varepsilon_{0}\left[{\left\{\tilde{n}\left(\omega \right)\right\}}^{2}-\varepsilon_{\infty }\right]$$

(5) $$\sigma^{\prime }=\omega \varepsilon_{0}\varepsilon^{\prime \prime },\;\sigma^{\prime \prime }=\omega \varepsilon_{0}\left(\varepsilon_{\infty }-\varepsilon^{\prime }\right)$$

where *ε*_0_ is the permittivity of free space; *ω* is the angular frequency; and $\varepsilon_{\infty }$ is the permittivity of material at infinite frequency. The solid fitted line plots of complex permittivity and complex optical conductivity were obtained from Drude–Lorentz model fitting, as follows [35,36,37].

(6) $${\tilde{\varepsilon }}_{DL}\left(\omega \right)=\varepsilon_{\infty }-\frac{\omega_{p}^{2}}{\omega^{2}+i\Gamma_{D}\omega }-\sum \frac{A_{L}}{\omega^{2}-\omega_{L}^{2}+i\omega \Gamma_{L}}$$

where *ω_p_* is the plasma frequency; Γ*_D_* is the Drude damping constant; $A_{L}$ is the Lorentz oscillator strength; *ω_L_* is the phonon frequency; and Γ*_L_* is the spectral width. The Drude and Lorentz model fitting parameters are presented in Table 1.

As shown in Figure 5a, real and imaginary parts of the complex permittivity of the GOQD material were observed to be higher than those of the rGOQDs, thus indicating that more oxygen functional groups in the GOQDs affected their dielectric properties. The Lorentz oscillator strength *A_L_*, which was determined using Drude–Lorentz model fitting and is related to the dielectric property, also shows the same results as shown in Table 1. The *A_L_* value of GOQDs was higher compared to that of rGOQDs. Furthermore, the carrier density of rGOQDs is approximately 1.5-times higher than that of GOQDs, as determined by the plasma frequency (*ω_p_* ∝ *N*^1/2^). The observed results were attributed to the presence of a larger number of oxygen functional groups bonded to the carbon atoms in GOQDs. However, it was observed that the optical conductivity of GOQDs was higher than that of rGOQDs, as shown in Figure 5b. This result can be attributed to the presence of numerous defects within the rGOQD structure, such as carbon atom absence resulting from thermal reduction. These defects disturb the movement of the charge carriers and reduce electron paths, thereby affecting the mobility of charge carriers (*μ* = e/m × Γ*_D_*). As presented in Table 1, rGOQDs had lower charge carrier mobility compared to GOQDs, indicating a decrease in the carrier mobility of rGOQDs due to the presence of defects, leading to a decrease in the optical conductivity of rGOQDs compared to GOQDs. Moreover, the shorter scattering time (1/Γ*_D_*) was also observed in rGOQDs compared with GOQDs. To confirm the extent of the degree of defects in both materials, Raman spectroscopy was conducted to analyze their crystalline characteristics.

**Figure 6:**
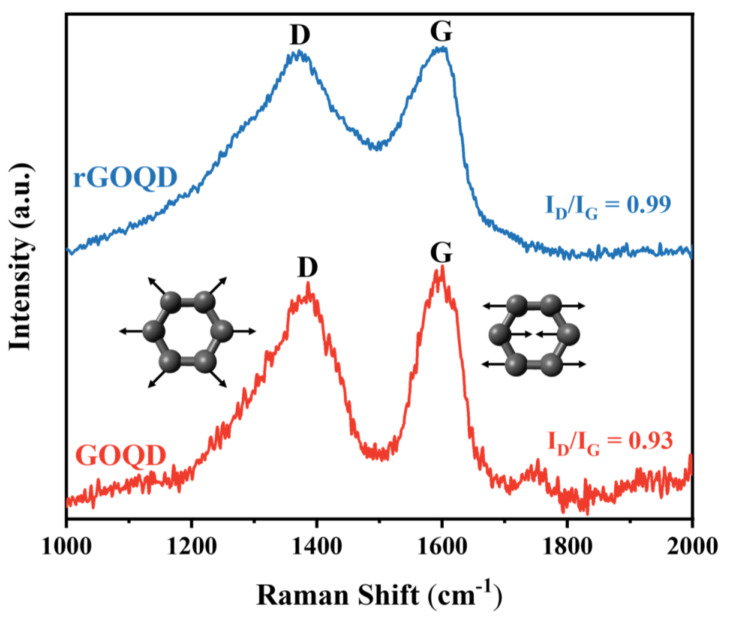
Raman spectra of GOQDs and rGOQDs.

Raman spectroscopy was carried out to analyze and characterize the defect states present in both GOQD and rGOQD samples by examining the vibrational modes and structural properties of the materials. The Raman spectra of both materials exhibited two characteristic peaks corresponding to the G and D bands. The two vibrational mode peaks D and G contributed to the structural defects and first-order scattering of the sp^2^ carbon, respectively. As shown in Figure 6, the intensity of the D band increased in the rGOQDs, thus revealing that the reduction process generated defects in the structure. The intensity ratios of the D and G peaks (I_D_/I_G_) were obtained to compare the degrees of disorder between GOQDs and RGOQDs. The I_D_/I_G_ ratios of the GOQDs and rGOQDs were 0.93 and 0.99, respectively, indicating a higher level of surface defects in the rGOQDs compared with the GOQDs [38,39,40]. As previously mentioned, a higher defect degree in the rGOQDs affects the charge carrier behaviors within the structure, thereby decreasing the optical conductivity [31,41]. The materials with lower crystallinity or a higher defect degree tend to exhibit reduced carrier mobility due to scattering and hindrances in charge transport. Therefore, the result indicates that the crystallinity of a material plays a crucial role in the carrier behavior such as carrier mobility.

## 4. Conclusions

In conclusion, GOQDs were synthesized using an oxidative cutting method with H_2_SO_4_, HNO_3_, and graphite as a precursor. Subsequently, rGOQDs were prepared via the thermal reduction process of GOQD. The sizes of the prepared samples were determined to be approximately 5 nm through TEM analysis. Furthermore, FT-IR and XPS were performed to verify the removal of oxygen functional groups in the rGOQD sample in comparison to GOQDs, demonstrating the successful reduction of GOQDs. To obtain the optical properties and analyze the carrier behaviors, THz-TDS was performed using the three free-standing GOQD and rGOQD pellets, which were fabricated under the same conditions. The measurements were conducted within an acrylic box filled with dry air to avoid the absorption of the THz signal by water vapor. Initially, the absorption coefficient and refractive index were obtained through THz-TDS results to determine the complex permittivity and optical conductivity of the prepared samples in the THz region. The obtained complex permittivity and optical conductivity were fitted using the Drude–Lorentz model, and the fitting parameters that demonstrate the carrier behaviors were acquired. As a result, the higher dielectric property and optical conductivity were confirmed in the GOQD sample. The more oxygen functional groups in the GOQDs were attributed to the increase in dielectric properties. In addition, more surface defects generated by the thermal reduction process in the rGOQDs were attributed to the decrease in optical conductivity. The higher disorder degree was investigated in the rGOQD structure compared with the GOQDs using Raman spectroscopy. Although a higher carrier density was obtained in the rGOQDs via Drude–Lorentz model fitting, the numerous surface defects within the rGOQD structure hindered the movement of charge carriers, thereby resulting in decreased conductivity and lower carrier mobility. Our work contributes to the advancement of knowledge and understanding of the optical properties and carrier behavior of GQDs, a promising and emerging class of nanomaterial. Moreover, to the best of our knowledge, this study is the first to provide insights into the factors influencing the carrier behavior within GQDs in various fields where carrier behavior plays a crucial role. Specifically, our study highlights the significant influence of defects within the GQD structure on carrier movements, emphasizing that carrier behavior is more affected by structural defects than the carrier density of the sample. Consequently, this finding is expected to contribute to the development of various fields where carrier behavior is crucial, such as solar cells and photocatalysis, by providing essential information on the factors influencing carrier behavior.

## Figures and Tables

**Table 1:** Drude-Lorentz oscillator model fitting parameters of GOQDs and rGOQDs. Plasma frequency *ω_p_*, Drude damping rate Γ*_D_*, Lorentz oscillator strength *A_L_*, phonon frequency *ω_L_*, spectral width Γ*_L_*, charge carrier density *N*, and mobility *μ*.

Sample	*ω_p_* [THz]	Γ*_D_* [THz]	*A_L_*	*ω_L_* [THz]	Γ*_L_* [THz]	*N* [10^15^ cm^−3^]	*μ* [cm^2^ V^−1^ s^−1^]
GOQD	2.55 ± 0.18	18.53 ± 0.76	1.42 ± 0.21	13.47 ± 0.79	11.64 ± 2.49	2.05 ± 0.29	256.80 ± 10.74
rGOQD	3.13 ± 0.13	20.71 ± 0.52	1.04 ± 0.26	12.57 ± 0.26	8.33 ± 0.75	3.07 ± 0.25	229.61 ± 5.88

## Data Availability

The data that support the findings of this study are available from the corresponding author upon reasonable request.
