# Correction: Song et al. Terahertz Optical Properties and Carrier Behaviors of Graphene Oxide Quantum Dot and Reduced Graphene Oxide Quantum Dot via Terahertz Time-Domain Spectroscopy. *Nanomaterials* 2023, *13*, 1948

**DOI:** 10.3390/nano15151164

**Published:** 2025-07-28

**Authors:** Seunghyun Song, Hyeongmun Kim, Chul Kang, Joonho Bae

**Affiliations:** 1 Department of Physics, Gachon University, 1342 Seongnamdaero, Sujeong-gu, Seongnam 13120, Republic of Korea; songsh139@gmail.com; 2 Department of Physics, Chonnam National University, 77 Yongbong-ro, Buk-gu, Gwangju 61186, Republic of Korea; u00extreme@naver.com; 3 Advanced Photonics Research Institute, Gwangju Institue of Science and Technology, 123 Cheomdangwagi-ro, Buk-gu, Gwangju 61005, Republic of Korea

In the original publication [1], there was a typographical mistake in Table 1 as published. The corrected Table 1 appears below.

The authors state that the scientific conclusions are unaffected. This correction was approved by the Academic Editor. The original publication has also been updated.

## Figures and Tables

**Table 1:** Drude-Lorentz oscillator model fitting parameters of GOQDs and rGOQDs. Plasma frequency *ω_p_*, Drude damping rate Γ*_D_*, Lorentz oscillator strength *A_L_*, phonon frequency *ω_L_*, spectral width Γ*_L_*, charge carrier density *N*, and mobility *μ*.

Sample	*ω_p_* [THz]	Γ*_D_* [THz]	*A_L_*	*ω_L_* [THz]	Γ*_L_* [THz]	*N* [10^15^ cm^−3^]	*μ* [cm^2^ V^−1^ s^−1^]
GOQD	2.55 ± 0.18	18.53 ± 0.76	1.42 ± 0.21	13.47 ± 0.79	11.64 ± 2.49	2.05 ± 0.29	256.80 ± 10.74
rGOQD	3.13 ± 0.13	20.71 ± 0.52	1.04 ± 0.26	12.57 ± 0.26	8.33 ± 0.75	3.07 ± 0.25	229.61 ± 5.88
